# Nutrients, herbal bioactive derivatives and commensal microbiota as tools to lower the risk of SARS-CoV-2 infection

**DOI:** 10.3389/fnut.2023.1152254

**Published:** 2023-06-01

**Authors:** Arianna Romani, Domenico Sergi, Enrico Zauli, Rebecca Voltan, Giada Lodi, Mauro Vaccarezza, Lorenzo Caruso, Maurizio Previati, Giorgio Zauli

**Affiliations:** ^1^Department of Environmental and Prevention Sciences and LTTA Centre, University of Ferrara, Ferrara, Italy; ^2^Department of Translational Medicine and LTTA Centre, University of Ferrara, Ferrara, Italy; ^3^Curtin Medical School & Curtin Health Innovation Research Institute (CHIRI), Faculty of Health Sciences, Curtin University, Perth, WA, Australia; ^4^Research Department, King Khaled Eye Specialistic Hospital, Riyadh, Saudi Arabia

**Keywords:** SARS-CoV-2, COVID-19, natural products, phytochemicals, ACE-2, TMPRSS2

## Abstract

The SARS-CoV-2 outbreak has infected a vast population across the world, causing more than 664 million cases and 6.7 million deaths by January 2023. Vaccination has been effective in reducing the most critical aftermath of this infection, but some issues are still present regarding re-infection prevention, effectiveness against variants, vaccine hesitancy and worldwide accessibility. Moreover, although several old and new antiviral drugs have been tested, we still lack robust and specific treatment modalities. It appears of utmost importance, facing this continuously growing pandemic, to focus on alternative practices grounded on firm scientific bases. In this article, we aim to outline a rigorous scientific background and propose complementary nutritional tools useful toward containment, and ultimately control, of SARS-CoV-2 infection. In particular, we review the mechanisms of viral entry and discuss the role of polyunsaturated fatty acids derived from α-linolenic acid and other nutrients in preventing the interaction of SARS-CoV-2 with its entry gateways. In a similar way, we analyze in detail the role of herbal-derived pharmacological compounds and specific microbial strains or microbial-derived polypeptides in the prevention of SARS-CoV-2 entry. In addition, we highlight the role of probiotics, nutrients and herbal-derived compounds in stimulating the immunity response.

## 1. Introduction

The coronavirus disease 2019 (COVID-19) pandemic, caused by severe acute respiratory syndrome coronavirus 2 (SARS-CoV-2), has triggered a devastating global health, social and economic crisis, with more than 664 million cases and 6.7 million deaths (1). Coronaviruses are a group of enveloped, positive-sense, single-stranded RNA viruses that are able to cause a range of diseases in several species including humans (2).

Several different strains of human coronaviruses (HCoV) have been identified to date (3). Among them, SARS-CoV, MERS-CoV, and SARSCoV-2 are highly pathogenic and have resulted in three life-threatening severe respiratory disease outbreaks in the past two decades. Other HCoV strains [i.e., HCoV-229E (an alpha CoV), HCoV NL63 (an alpha CoV), HCoV-OC43 (a beta CoV), and HCoV HKUI (a beta CoV)] usually cause only common-cold-like mild upper respiratory tract illnesses in humans (3, 4). As these human coronaviruses have a zoonotic origin, it is increasingly likely that there will be more HCoV outbreaks in the future (5). The envelope spike (S) protein of SARS-CoV-2 plays a crucial role in coronavirus pathogenesis, mediating receptor binding, membrane fusion and promoting viral entry into target cells (6). The S protein of coronaviruses is functionally divided into the S1 domain, the receptor binding domain (RBD), and the S2 domain responsible for cell membrane fusion. Virus entry requires S protein priming by cellular proteases which determine the cleavage of S1/S2 domains and allow fusion of the viral envelope with the cellular membrane. SARS-CoV-2 engages angiotensin-converting enzyme 2 (ACE2) as the entry receptor and uses the cellular transmembrane serine protease 2 (TMPRSS2) for S protein priming. ACE2 and TMPRSS2 are expressed in several human cells, including cells of the respiratory and digestive tracts (7, 8).

Original data suggest that the downregulation of TMPRSS2 and/or ACE2 expression on the cell surface could avert viral entry into the host cell and, consequently, infection spreading. It has been shown that knocking out ACE2 expression can block SARS-CoV-2 infection of murine epithelial cells (9), and that ACE inhibition blocks SARS-CoV-2 infection *in vitro* (10, 11).

This review is focused on natural products, including nutrients, bacterial strains, molecules or herbal extracts that target virus entry by interfering with ACE2 binding and/or by preventing TMPRSS2 cleavage. In addition, considering the hyperinflammation rise often associated with SARS-CoV-2 infection (12), we highlight also the anti-inflammatory properties associated with several natural products.

## 2. Virus structure, biology

The RNA genome of SARS-CoV-2 (29.9 kb) encodes 29 proteins (13). Of these, only four proteins, namely S protein, membrane (M), envelope (E), and nucleocapsid (N), make up the whole virus structure (14). The remaining proteins are non-structural proteins (*n* = 16) and accessory proteins (*n* = 9) that are pivotal in the replication of the virus and the escape of host immunity (15). Similar to SARS-CoV, SARS-CoV-2 utilizes the cell surface receptor ACE2 for cellular entry. Firstly, the SARS-CoV-2 S glycoprotein interacts with surface ACE2 to enter the target cell; in addition, invasion also needs proteolytic activation of the S protein, which is helped by the TMPRSS2 and lysosomal cysteine proteases, cathepsins, available in the target host cell (7, 8). Newly made envelope proteins are inserted into endoplasmic reticulum and Golgi membranes, and the nucleocapsid is formed by the assimilation of nucleocapsid protein with genomic RNA. Then, viral particles are produced into the endoplasmic-reticulum-Golgi intermediate compartment and the virus particles are released by exocytosis. Any of the steps in this viral life cycle are a potential target for anti-SARS-CoV-2 drug discovery.

To rationally target the SARS-CoV-2 life cycle, it is important to better outline at least the more general steps of virus entry. The S protein of SARS-CoV-2 is not only the main mediator of initial virus attachment on the cell surface, but also ignites the complex machinery that allows viral RNA entry into the host cytoplasm by triggering pore formation, both during membrane fusion and endocytosis. Similarly to HIV-1 and Ebola viruses, the preliminary step for S protein priming occurs in the infected cells, during the production of new viral particles (16). During this stage, cellular proteases like furin cleave S proteins into two not-covalently-associated S1 and S2 subunits. The S1 subunit is responsible for the attachment to the obligate SARS-CoV-2 receptor, the ACE2 protein, which in the human body is expressed at high levels in the small intestine, testis, kidney, heart muscle, colon and thyroid gland (17). When the viral and the host cell membrane are proximal, the docking of S1 to ACE2 determines ACE2-dependent conformational changes at the S2 sequence. From this moment, TMPRSS2 becomes pivotal in determining the modalities of virus entry. Sufficient expression or activity of TMPRSS2 at cell surface level allows for S2 cleavage, exposure of fusion peptide sequences, followed by the process by which enveloped viruses merge their membrane with the host cell membrane in a way that the virus can move its genome inside the cell, resulting in the potential production of new virions (18). Of note, membrane fusion is not a spontaneous process, as there are high energy requirements to bring the membranes close together (19, 20). Alternatively to membrane fusion, SARS-CoV-2 can take advantage also from endocytic pathways to reach the cytosol of the host cell. In the absence of S1 priming, the virus binding to one or more copies of ACE2 can trigger the endocytic route. After internalization, and presumably at the stage of late endosome, cathepsin protease activity on S2 determines the exposure of S2 sequences which allow the fusion of viral and endosomal membranes, followed by liberation of viral RNA into the cytoplasm. Interestingly, not only naïve but also opsonized virions can be internalized by the endocytic mechanism. Opsonized SARS-CoV-2 virions are coated with antibodies that mask viral proteins, but the virus can still bind the cell surface and be endocytosed due to the presence of receptors for antibody Fc regions (21).

Of note, as SARS-CoV-2 circulated globally, the viral genome acquired new mutations, some of which have become widespread. Until late 2020, the most notable was the S protein mutation D614G. This variant quickly became dominant, and this rapid spread seems to have been due to increased infectivity, stability, and transmissibility over the ancestral D614 form (22, 23), resulting from a shift to the open configuration of the S protein trimer, which is required for binding to the host ACE2 receptor (23) and host cell entry. Not surprisingly, there are many variants of SARS-CoV-2. Some are believed or have been stated to be of particular importance, due to their potential for increased transmissibility (24), increased virulence, or reduced effectiveness of vaccines against them (25, 26). Studies have demonstrated reductions in neutralizing activity of vaccine-elicited antibodies against a range of SARS-CoV-2 variants, against the Omicron variants in particular, exhibiting partial immune escape. However, evidence suggests that T-cell responses are preserved across vaccine platforms, regardless of the variant of concern (26, 27). As of March 2023, only the Omicron variants are designated as a circulating variant of concern by the World Health Organization (28).

Mechanistic details of these pathways may vary considerably between cell types. Regarding SARS-CoV-2’s most important cell target, it is worth noting that the diversity of endocytosis in airway epithelium is currently poorly understood. Dissecting the mechanisms of endocytic viral entry in the respiratory tract may therefore offer a promising therapeutic strategy to treat viral infections.

## 3. COVID-19 pathogenesis and pathophysiology

The SARS-CoV-2 virus is able to infect a wide range of cells and organs of the body. SARS-CoV-2 is most known for affecting the upper respiratory tract (sinuses, nose, and throat) and the lower respiratory tract (bronchi and lungs). The lungs are mostly affected by SARS-CoV-2 because ACE2 is most abundant on the surface of type II alveolar pneumocytes of the lungs (29, 30). Three common patterns of symptoms have been recognised: one respiratory symptom cluster with cough, sputum, shortness of breath, and fever; a musculoskeletal symptom cluster with muscle and joint pain, headache, and fatigue; a cluster of digestive symptoms with abdominal pain, vomiting, and diarrhea (31).

Genetic predisposition may have a role in COVID-19 pathogenesis. The receptor-binding domain (RBD) of the SARS-CoV-2 S protein binds with high affinity with ACE2 receptor to enter cells. Consequently, ACE2 genetic variants that could affect its gene expression, protein conformation, and protein stability are the one of most uncertain factors involved genetic predisposition to SARS-CoV-2 infection (32). ACE2 is an X-linked gene that harbors a strong variant with tendency to an X-linked dominant inheritance pattern in severely affected patients. It might be a clue to the reason for the higher prevalence and severity of COVID-19 in men than in women (33). Furthermore, the immune-related genetic variants associated with the prior strain of coronavirus, namely SARS-CoV, are suspected to have roles in the genetic predisposition to SARS-CoV-2 infection (34), since SARS-CoV-2 has 80% genetic identity to SARS-CoV (35). Focusing on the genes of the human immune system and relating them to SARS-CoV-2 susceptibility, several lines of evidence strongly support the role of the interferon system (and related cytokines) as the most important determinant of infection control versus infection severity in humans (36–38).

### 3.1. COVID-19 and calcium metabolism

An important issue in the pathogenesis of SARS-CoV-2 infection is the role of calcium signaling. Of note, at the cellular level, coronavirus infection has been shown to modulate calcium metabolism. The SARS-CoV-2 S protein has two FP domains, FP1 and FP2, and binds to two Ca^2+^ ions for host cell entry (39). SARS-CoV-2 appears to affect cellular function by altering the host Ca^2+^ homeostasis in ways that promote viral infection and reproduction. One mechanism is through disruption of calcium channels and pumps (e.g., voltage-gated calcium channels (VGCCs), receptor-operated calcium channels, store-operated calcium channels, transient receptor-potential ion channels, and Ca^2+^-ATPase) (40). Furthermore, the E and ORF3a proteins of coronaviruses impact Ca^2+^ homeostasis in the host, by acting as calcium ion channels, enhancing the virion’s entry and replication potential (41).The SARS-CoV-2-E protein is a 76-amino-acid (aa) integral membrane protein with one transmembrane domain (TMD) that allows the E protein to form protein-lipid channels in membranes that promote permeability to Ca^2+^ ions. The alteration of Ca^2+^ homeostasis by SARS-CoV-2 proteins promotes SARS-CoV-1/2 fitness and elicits the production of chemokines and cytokines, contributing to pathogenesis. Ion channel activity modulation by the SARS-CoV-1-ORF3a protein also modulates viral release (42). Therefore, when SARS-CoV-2 infects the human body, the resultant dysregulation of Ca^2+^ homeostasis may contribute to morbidity and mortality. COVID-19 patients have been noted to have low serum calcium levels overall (43).

### 3.2. COVID-19 and oxidative balance

In addition to calcium homeostasis alteration, imbalance between oxidative species and antioxidants also has a proven role in COVID-19 pathogenesis. The presence of oxidative stress in COVID-19 patients was recently assessed in studies that observed a significant reduction in free sulfhydryl groups from patient serum (44), and a mortality-related increase in damaged albumin (45). In addition, several other markers like glial fibrillary acidic protein (GFAP), the receptor for advanced glycation end products (RAGE), high mobility group box-1 protein (HMGB1) and cyclo-oxygenase-2 (COX-2) were found increased in patients with severe COVID-19 (46). Another study outlined, in COVID-19 patients, inflammasome activation correlated with mitochondrial superoxide and lipid peroxidation, suggesting that oxidative stress and inflammation are two sides of the same coin, where inflammation and oxidative stress reinforce each other (47). In particular, high reactive oxygen species (ROS) levels, originating from improper oxidative metabolism and the action of defensive enzymes such as NADPH oxidase, could lead to the formation of oxidized forms of proteins, DNA and lipids that in turn could act as damage-associated molecular patterns (DAMPs) which could trigger further inflammatory reaction (48), ultimately unbalancing antiviral response and inflammation regulation, to unleash a dysregulated cytokine production normally known as cytokine storm (49). So, the pathological oxidative response, followed by reduced nitric oxide production and increased endothelial dysfunction, hyperpermeability and hypercoagulability, leads to a scenario of hyperinflammation and thrombosis, that, together with immunosuppression, constitute the core of COVID-19 disease.

### 3.3. Host cytokine response

Subjects with severe COVID-19 have symptoms of systemic hyperinflammation and dysregulated immune response. Laboratory findings of increased interleukin-2 (IL-2), interleukin-7 (IL-7), interleukin-6 (IL-6), granulocyte-macrophage colony-stimulating factor (GM-CSF), C-X-C Motif Chemokine Ligand 10 (CXCL10), monocyte chemoattractant protein-1 (MCP1), macrophage inflammatory protein-1 α (MIP1α), and tumor necrosis factor α (TNF-α) are indicative of cytokine release syndrome and are suggestive of an underlying immunopathology (36, 50–52). The severity of the inflammation can be linked to the severity of what is known as the cytokine storm. Combatting the cytokine storm has been proposed as an effective treatment since it is one of the leading causes of morbidity and mortality in COVID-19 (36, 53, 54). A cytokine storm is caused by an acute hyperinflammatory response that is responsible for clinical illness in an array of diseases; and in COVID-19, it is related to a worse prognosis and increased fatality. The storm causes acute respiratory distress syndrome and blood clotting events such as thrombosis, strokes, myocardial infarction, encephalitis, acute kidney injury, and vasculitis. The production of IL-1, IL-2, IL-6, TNF-α, and interferon-gamma (IFN-γ), all crucial components of normal immune responses, become the causes of a cytokine storm.

In addition, key transcriptional factors, such as tumor protein 53 (p53) and nuclear factor kappa-light-chain-enhancer of activated B cells (NF-kB) and their reciprocal balance, are altered upon SARS-CoV-2 infection (55). Of note, interferon alpha (IFN-α) plays a complex, multi-faceted role in the pathogenesis of COVID-19. Although it promotes the elimination of virus-infected cells, it also upregulates the expression of ACE2, thereby facilitating the SARS-CoV2 virus to enter cells and replicate. A competition of negative feedback loops (via protective effects of IFN-α) and positive feedback loops (via upregulation of ACE2) is assumed to determine the fate of patients suffering from COVID-19 (37, 56, 57). Additionally, subjects with COVID-19 and acute respiratory distress syndrome (ARDS) have classical serum biomarkers of cytokine release syndrome, including elevated C-reactive protein lactate dehydrogenase D-dimer and ferritin levels (36, 58). Systemic inflammation results in vasodilation, allowing inflammatory lymphocytic and monocytic infiltration of the lung and the heart. Of note, pathogenic GM-CSF-secreting T cells were linked to the recruitment of pro-inflammatory IL-6-secreting monocytes and severe lung pathology in COVID-19-infected subjects (36). Wide-spread lymphocytic infiltrates have also been reported at autopsy (59).

### 3.4. COVID-19 and the central nervous system

Loss of smell, a common symptom, results from infection of the cells of the olfactory epithelium, with subsequent damage to the olfactory neurons. The involvement of both the central and peripheral nervous system in COVID-19 has been reported (60, 61). The virus is not detected in the central nervous system (CNS) of the majority of COVID-19 patients with neurological issues. However, SARS-CoV-2 has been detected at low levels in the brains of those who have died from COVID-19, but these results need to be confirmed (60, 61). While the virus has been detected in cerebrospinal fluid in autopsies, the exact mechanism by which it invades the CNS remains unclear, and it could involve invasion of peripheral nerves due to the low expression levels of ACE2 in the brain (60, 61). The virus may also enter the bloodstream from the lungs and cross the blood–brain barrier to gain access to the CNS, possibly within infected white blood cells (61). Observed individuals infected with SARS-CoV-2 (most with mild cases) experienced an additional 0.2–2% of brain tissue lost in regions of the brain connected to the sense of smell compared with uninfected individuals; infected individuals also scored lower on several cognitive tests. All effects were more pronounced among elderly individuals (61).

### 3.5. COVID-19 and the gastrointestinal tract

The virus also involves gastrointestinal (GI) organs, since ACE2 is expressed in the glandular cells of gastric, duodenal and rectal epithelia as well in the enterocytes of the small intestine (62). Potential mechanisms on how SARS-CoV-2 can cause damage to the GI tract include a direct virus-induced cytopathic effect through cell entry via ACE2, indirect immune-mediated injury triggered by a systemic inflammatory response to SARS-CoV-2, and disruption of the intestinal microecological “milieu” leading to excessive systemic inflammation which may lead to a cytokine storm. Of particular interest, in our opinion, is the role of direct mucosal damage and the role of the intestinal microbiota. SARS-CoV-2 infection of gut epithelial cells is able to trigger dysbiosis, intestinal inflammation, and GI symptoms (63). The cytopathic viral effect on target intestinal cells leads to the generation of inflammatory signals known as pathogen-associated molecular patterns (PAMPs) and intracellular DAMPs, which stimulate pattern recognition receptors (PRRs) such as toll-like receptors (TLRs), retinoic acid-inducible gene I (RIG-I) and other RIG-I-like receptors (RLRs). DAMPs and PAMPs trigger, through the recruitment of specific adaptors, the innate immune response which implicates the production of cytokines and chemokines such as TNF-α, interleukin-1 beta (IL-1β), IFNs, IL-6, CXCL10, MIP1α, MIP1β and MCP1.

### 3.6. COVID-19 and the cardiovascular system

Additionally, the virus can cause acute myocardial injury and chronic damage to the cardiovascular system. An acute cardiac injury was found in 12% of infected people admitted to the hospital in Wuhan, China, and it is more frequent in severe disease. Rates of cardiovascular symptoms are high, in accordance with the systemic inflammatory response and any immune system disorders during disease progression. However, acute myocardial injuries may also be related to the high expression of ACE2 receptors in the heart (64).

A high incidence of thrombosis and venous thromboembolism occurs in people transferred to intensive care units with SARS-CoV-2 infections and may be related to poor prognosis. Blood vessel dysfunction and clot formation (as suggested by high D-dimer levels caused by blood clots) are likely playing a significant role in mortality, with the incidence of clots leading to pulmonary embolisms, and ischaemic events within the brain (found as complications) leading to death in people infected with SARS-CoV-2. Infection may trigger a chain of vasoconstrictive responses within the body, including pulmonary vasoconstriction, decreasing oxygenation. Moreover, microvascular and capillary damage was found in the brain tissue of people who died from COVID-19 (65–67).

### 3.7. COVID-19 and blood cells changes

SARS-CoV-2 is also able to cause structural changes to blood cells, in some cases persisting for months after hospital discharge. A low level of blood lymphocytes may result from the virus acting through ACE2-related entry into lymphocytes.

One of the most notable changes seen in patients with COVID-19 is the alteration in their blood cell counts (68). During SARS-CoV-2 infection, there is a decrease in the number of white blood cells, particularly lymphocytes. The decrease in lymphocyte count is associated with the severity of the disease, and patients with severe COVID-19 tend to have lower lymphocyte counts (52). Of note, patient’s T cell compartment shows several alterations involving naïve, central memory, effector memory and terminally differentiated cells, as well as regulatory T cells and PD1 + CD57+ exhausted T cells (52). T cells exhibit indications of exhaustion, such as increased expression of inhibitory receptors like PD-1. This state of exhaustion is marked by functional unresponsiveness, which serves to prevent extensive immune activation and the resultant tissue damage from autoimmune reactions. As a result, it is plausible that activation of these cells in COVID-19 patients not only results in a lack of clonal expansion, as evidenced by decreased proliferation, but also leads to the production of molecules that promote inflammation (52). The levels of immunoglobulin classes and antibodies against common antigens or vaccines in COVID-19 patients’ plasma were found to be normal. However, the number of total and naïve B cells decreased, along with decreased percentages and numbers of memory switched and unswitched B cells. Conversely, there was a significant increase in IgM+ and IgM-plasmablasts. B lymphocytes showed normal proliferation index and number of dividing cells per cycle during *in vitro* cell activation. The principal component analysis (PCA) indicated that B-cell number, naïve and memory B cells, but not plasmablasts, clustered with patients who were discharged. On the other hand, plasma IgM level, C-reactive protein, D-dimer, and sequential organ failure assessment (SOFA) score clustered with those who died. In patients with pneumonia, the deterioration of the B-cell compartment could be one of the reasons for immunological failure in controlling SARS-CoV2 (52). During SARS-CoV-2 infection, there is a decrease in the number of red blood cells, leading to anemia (69). Anemia can cause fatigue, shortness of breath, and other symptoms. The decrease in red blood cell count is also associated with the severity of the disease (69). Overall, structural and functional alterations of the blood cell compartment have an important role in the pathogenesis of SARS-CoV-2 infection; recent data highlight the predictive role of these alterations in prognosis and in the long COVID clinical setting (70–72).

## 4. The role of nutrients in preventing SARS-CoV-2 infection

### 4.1. Dietary omega-3 fatty acids as a tool to prevent COVID-19

The nutritional status of the host represents a pivotal discriminant influencing the ability of SARS-CoV-2 to enter cells and replicate. In this regard, dietary nutrients are emerging as a potential modulator of SARS-CoV-2 infections. Of these, bioactive fatty acids, like omega-3, may play a role in this context. Omega-3 are polyunsaturated fatty acids derived from α-linolenic acid which represents the precursor of eicosapentaenoic (EPA) and docosahexaenoic acid (DHA). While α-linolenic acid is an essential fatty acid, and therefore can only be obtained from the diet, EPA and DHA can be endogenously synthesised from the mutual precursor or obtained mainly via the consumption of fatty fish or fish oil. Regarding their role as potential nutraceuticals to tackle the COVID-19 pandemic, the supplementation of omega-3 fatty acids has been associated with a lower risk of SARS-CoV-2 infection, at least in women (73). This is supported by the ability of these polyunsaturated fatty acids to protect against viral infections by inhibiting viral entry, localization and replication (74). The impact of omega-3 fatty acids on viral entry into the cells is dictated by their capacity to modulate membrane fluidity and protein complex formation in lipid rafts. In turn, the entry gateway for SARS-CoV-2, ACE2 and TMPRSS2, is most commonly found in lipid rafts (75). Additionally, the size and number of lipid rafts may impact the abundance as well as enzymatic activity of ACE2 and TMPRSS2 (74). Thus, the modulation of lipid rafts by omega-3 fatty acids may affect viral entry into the cells (76). Furthermore, polyunsaturated omega-3 fatty acids interfere with the virus binding to ACE2, with linolenic acid and EPA significantly blocking the entry of SARS-CoV-2 (77).

Aside from representing a nutritional tool potentially inhibiting SARS-CoV-2 entry into the cells, omega-3 fatty acids may also interfere with virus-mediated activation of sterol regulatory element binding proteins (SREBPs) which in turn are pivotal for viral replication. They facilitate viral replication, and modulate cellular lipid metabolism, leading to increased availability of lipid substrates directed towards virion replication membrane formation. Considering the central role of SREBP 1/2 in promoting lipogenesis and its involvement in the virus-mediated rewiring of lipid metabolism, this transcription factor has been proposed as a broad-spectrum anti-viral target (78). Not surprisingly, omega-3 fatty acids have been widely reported to influence lipid metabolism (79), an effect that also relies on their ability to inhibit SREBP1 activation and downregulate SREBP1c (80). Considering this, omega-3 may hinder virus-induced SREBP activation, thereby interfering with viral replication. Additionally, the regulation of cholesterol metabolism by SREBP may represent an additional mechanism explaining the potential role of omega-3 fatty acids in inhibiting SARS-CoV-2 infection. Indeed, cholesterol is also a key component of lipid rafts, and as such, it is crucial in mediating the entry of the virus into the cells (81). Thus, it appears that the ability of omega-3 fatty acids to counter SARS-CoV-2 infection relies on their impact upon lipid metabolism. This possibility is further supported by the fact that lipogenesis modulator drugs, able to hamper fatty acid and cholesterol synthesis, also alter SARS-CoV-2 replication cycle *in vitro* (82). In light of this, the therapeutic potential of lipogenesis modulators against COVID-19 may also apply to nutrients able to affect lipid metabolism, such as omega-3 which may therefore represent a promising nutritional tool to tackle SARS-CoV-2 infection. Despite this, direct evidence gathered through clinical trials, on the ability of omega-3 fatty acids to prevent or at least limit SARS-CoV-2 infection, is still lacking.

Another potential mechanism by which omega-3 fatty acids may interfere with SARS-CoV-2 infection is via the modulation of the activity of the immune system. Particularly, the fatty acid composition of the plasma membranes of phagocytic cells modulates their phagocytic capacity (83), which in turn represents an important step in the immune response against foreign pathogens. In support of this, while omega-3 fatty acids have the potential to enhance the phagocytic activity of neutrophils and monocytes, this effect is negatively correlated with palmitic acid content in the plasma membrane of these cells [84; 85]. Considering that the fatty acid composition of cell membranes closely reflects dietary fatty acid intake, it is plausible that increasing the intake of omega-3 fatty acids may result in an increase of phagocytic activity. Indeed, the supplementation of DHA and EPA at a dose of 1.5 g/day results in an increase in the phagocytic activity of both neutrophils and monocytes (84, 85).

Another mechanism by which omega-3 fatty acids, but also other unsaturated fatty acids, may dampen viral infection is via the disruption of the virus envelope. Aside from the aforementioned unsaturated fatty acids, this effect is also elicited by medium-chain fatty acids, while short-and long-chain saturated fatty acids do not show anti-viral activity against enveloped viral particles (86).

Additionally, apart from their ability to potentially combat SARS-CoV-2 infection, omega-3 fatty acids may also play a role in dampening the severity of the health complications secondary to the infection (74). Omega-3 fatty acids, given their anti-inflammatory role, represent a valuable aid in countering COVID-19-induced inflammation and therefore prevent the cytokine storm (87). However, these effects are not within the scope of this review, as they are not directly linked with the ability of these fatty acids to hinder SARS-CoV-2 infection and are reviewed elsewhere (74).

### 4.2. Vitamins and minerals as tools to mitigate the risk of SARS-CoV-2 infection

Vitamin and mineral status, and consequently their intake, are crucial for the host to mount effective defence responses against COVID-19. In this regard, vitamin D has been proposed as a putative preventative or therapeutic nutritional tool in the battle against SARS-CoV-2 infection (88). This paradigm is also supported by the fact that low levels of 25-hydroxyvitamin D3 have been associated with increased susceptibility to acute respiratory tract infections (89). In line with this, emerging evidence points to vitamin D as a potential nutritional tool able to lower the risk of SARS-CoV-2 infection and improve disease outcomes. Indeed, vitamin D supplementation was associated with a 9% decrease in the risk of SARS-CoV-2 infection, an effect that, as described for omega-3 fatty acids, was specific to females (73). On the contrary, low vitamin D status has been associated with a higher susceptibility to SARS-CoV-2 infection (90). From a mechanistic perspective, these effects may be dependent on the ability of vitamin D to support innate antiviral immune responses, including the induction of autophagy and the production of antimicrobial components of the innate immune system, such as cathelicidin (91). In further support of the antiviral effects of vitamin D, its active form, calcitriol, has shown an inhibitory effect against SARS-CoV-2 infection in an *in vitro* model of human nasal epithelial cells (92). Furthermore, vitamin D supplementation, aside from lowering the incidence of the infection, may decrease the severity of the symptomatology as well as the risk of death from COVID-19 (93). Nevertheless, despite the potential benefit of vitamin D supplementation in mitigating the risk of SARS-CoV-2 infection, also supported by the protective effects of this vitamin against acute respiratory infections (94), the data generated up to date do not infer a cause-effect relationship between vitamin D intake and prevention of SARS-CoV-2 infection (88). Additionally, the supplementation of cod oil providing 10 μg of vitamin D daily did not affect the incidence of SARS-CoV-2 infection (95), supporting the possibility that vitamin D supplementation, at least at the dosage provided as part of this study, may not be sufficient to mitigate the risk of SARS-CoV-2 infection. Thus, despite observational studies supporting the role of vitamin D in lowering the risk of SARS-CoV-2 infection (73, 96), the evidence gathered to date do not imply a direct causality between vitamin D status and lower SARS-CoV-2 infection incidence.

Vitamin E may also represent a potential molecule to fight off SARS-CoV-2 infection, as demonstrated by the relationship between α-tocopherol supplementation and the decrease in upper respiratory tract infection (97). The antiviral effects ascribed to vitamin E may be dependent upon its capacity to increase the number of T cells, and their ability to produce IL-2 and enhance the activity of natural killer cells (98). Despite its immunomodulatory potential, possibly involved in lowering the risk of SARS-CoV-2 infection, direct evidence of the role of vitamin E in preventing SARS-CoV-2 infection is still lacking.

Vitamin C is well known for its immune-boosting effects, and as such represents another micronutrient with the potential to lower the risk of SARS-CoV-2 infection. In this regard, vitamin C has been shown to elicit antiviral immune responses underlaid by an increase in IFN-α/β as demonstrated in the early stages of influenza virus infection (99). The antiviral effects exerted by vitamin C also relay on the upregulation of natural killer cells and the induction of cytotoxic T-lymphocyte activity (100, 101). Moreover, supplementation of vitamin C at doses of 1-2 g/day was effective in lowering the risk of upper respiratory tract infections (102). It is not surprising indeed that the highest rate of SARS-CoV-2 infection affected low-middle income countries where there is also a high prevalence of hypovitaminosis C (103), suggesting a putative relationship between vitamin C status and SARS-CoV-2 infection. In further support to the role of vitamin C in the battle against COVID-19, the deficiency of this vitamin has been reported in patients suffering from respiratory infections and patients with pneumonia, relative to healthy controls (104). Interestingly, there is an overlapping between vitamin C deficiency and many risk factors for SARS-CoV-2 infection and severity. Indeed, African-Americans, individuals affected by diabetes, hypertension and chronic obstructive pulmonary disease, not only are at high risk of developing severe, life-threatening symptoms due to SARS-CoV-2 infection, but also experience vitamin C deficiency (105). Moreover, scurvy, the direct consequence of vitamin C deficiency, is associated with defective immune function and increased susceptibility of infections like pneumonia (100, 106). Thus, the rationale for using vitamin C as a nutritional strategy to mitigate SARS-CoV-2 infection risk, is supported by the role of this vitamin as an immune-booster. Additionally, the anti-inflammatory, anti-oxidant and anti-thrombotic effects of this vitamin provide the rationale for its use to decrease the severity of the symptomatology in patients. This notion is supported by fact that individuals with lower serum vitamin C are at higher risk of severe COVID-19 (104), whereas intravenous administration of vitamin C in critically ill COVID-19 patients improved the symptomatology, lowered IL-6 circulating levels (107) potentially countering the cytokine storm, decreased mortality (104) and shortened the stay in the intensive care unit (108). Thus, vitamin C may represent a valuable tool not only to prevent the complications of the infection, but also to dampen the risk of SARS-CoV-2 infection given its antiviral and immunomodulatory properties (109). However, despite its immune boosting effects, which make it promising for primary prevention of viral infections, the direct relationship between vitamin C status and risk of SARS-CoV-2 infection remains to be fully elucidated.

Along with vitamins, some minerals have also been implicated in the prevention of SARS-CoV-2 infection. Of these, zinc may play a crucial role in this context, given its role in antiviral immunity (110). Zinc exploits an immunomodulatory role, as it regulates inflammatory responses, and the proliferation, differentiation and function of leucocytes and lymphocytes; it also promotes the secretion of IFN-α and-γ by leucocytes (111–113). In further support, its deficiency leads to decreased natural killer cell activity and impaired cytokine production by monocytes (114). Furthermore, zinc antiviral effects also rely on the inhibition of virus-host cell interaction as well as viral replication (110). Zinc, in a concentration-dependent fashion, also dampens ACE2 activity, possibly inhibiting its interaction with the SARS-CoV-2 S protein (115). High intracellular zinc concentration or agents able to enhance intracellular zinc influx inhibit the replication of several RNA viruses (116), which may also include SARS-CoV-2, possibly via inhibition of viral RNA polymerase activity (110). However, the latter effect has only been demonstrated on SARS-CoV *in vitro* (116). The role of zinc in supporting the immune system may also have implications for SARS-CoV-2 infection. In support of this, in a case–control study, symptomatic COVID-19 was significantly lower in individuals receiving zinc supplementation compared to controls (i.e., individuals not receiving zinc supplementation) (117). This suggests that maintaining an adequate zinc status may be instrumental in preventing and mitigating the severity of COVID-19 symptomatology (117). Not surprisingly, indeed, zinc deficiency was associated with acute respiratory distress syndrome and higher mortality rates (118). Thus, the prevention of zinc deficiency represents a promising strategy to support the immune system and possibly prevent the deleterious health consequences linked with COVID-19. However, as reported for the aforementioned nutrients, direct clinical evidence on the ability of zinc to counter SARS-CoV-2 infection is still lacking, which highlights the need for a prudent approach in the supplementation of zinc as a prophylaxis or treatment of COVID-19 (119).

## 5. Microbiome and COVID-19

### 5.1. Lung and gastrointestinal microbiome

The positive effect of probiotics on the human health is part of the more general interaction between human beings and the microorganisms populating the surfaces of the external part of the body or the internal cavities. At the intestinal level, the microbiota in a healthy individual is represented by an enormous number of microorganisms, consisting of bacteria, fungi, viruses, archaea and protozoa. Bacteria are represented by five major phyla, namely*, Firmicutes, Bacteroidetes, Actinobacteria, Verrucomicrobia, and Proteobacteria*. In the gut, the taxonomic composition, in terms of different genus and species and of their relative abundances, displays considerable variability among individuals (120).

A resident microbiota is present also in the respiratory tract, although the lower part was formerly believed to be sterile. Here, as reported by Magryś et al. (121), the microbial community is mainly represented by *Bacteroides, Firmicutes* and *Proteobacteria*, but the overall microorganism number is enormously reduced with respect to the intestinal bacterial population, and the species diversity is lower too.

The GI resident microbiota exerts several outstanding functions on human health. Generally, the commensal microorganisms participate in digestive processes, provided that hydrolytic enzymes are able to complete the demolition of otherwise non-digestible foods, and they are a source of several nutrients essential for the host, like vitamins and other nutrients (see below). In addition to the nutritional contribution, resident microorganisms exert both direct and indirect protective roles against exogenous or endogenous opportunistic pathogens integrating the host’s natural defenses.

Both intestinal and lung lumina are, in fact, hostile environments for both commensal and pathogen microorganisms. Goblet cells in ciliary and intestinal epithelia secrete a mucus layer, which interferes with the attachment of microorganism populations, and is further enriched by immunoglobulin A (IgA) secretion. In the gut, Paneth cells are characteristic epithelial elements that produce bactericidal substances like lysozyme, secretory phospholipase A2, defensins, defensin-like peptides, and cathelicidins (122). Altogether, mucus and bactericidal substances control and sharpen the survival of microorganisms at the epithelial surface and constitute an actual barrier defending underlying tissues from the aggression of pathologic microorganisms including viruses.

The microorganisms composing the customary commensal microbiome integrate passive host immunity defenses in several ways. First, they limit the adhesion and growth of pathogens to the epithelium surface, because commensals occupy the potential adhesion niches, competing for nutrients, and producing waste metabolites able to interfere with pathogen growth. Moreover, microorganisms continuously stimulate the production of mucus, IgA secretion and integrity of intraepithelial adhesion structures, which represent the first line of immunity defense against pathobionts.

Remarkably, the resident microorganisms entertain continuous crosstalk with the host immunity system which receives continuous stimulation. Members of innate and adaptive immunity, like alveolar or GI macrophages, dendritic cells, and regulatory T cells, are continuously challenged, until they reach a homeostatic state of interactive equilibrium. Importantly, this equilibrium is influenced by the reciprocal interaction between the lung and GI tract, and relative microbiome. This interaction, termed as the gut-lung axis, implies a reciprocal communication that occurs in different ways. Both organs can prime the local immune system whose components can be exchanged trough lymphatic and circulatory vessels. Mainly through the same routes, whole bacteria, bacterial fragments and microbiome-derived metabolites can be exchanged, making it possible to stimulate the immune system in diverse anatomical districts.

Of note, GI microorganisms release a wide number of metabolites including metabolized bile salts, short-chain fatty acids (SCFA), branched-chain amino acids, trimethylamine N-oxide, tryptophan and indole-derivative metabolites and imidazole propionate (123). Among them, SCFAs, (i.e., acetate, lactate, propionate, and butyrate) represent the end products of fermentative microbial metabolism, and at the same time, an important source of energy for colonocytes and a relevant caloric integration for the whole body. More importantly, SCFAs regulate immune cell division and metabolism in peripheral regulatory T cells, macrophages and granulocytes, antigen presentation by dendritic cells, and interleukin and cytokine production, often at distant locations (124–126). SCFAs can regulate genomic transcription, by both binding to specific free fatty acid receptors (FFARs) and inhibiting histone deacetylases (127, 128).

The importance of this stimulation by the microbiome on the immune system is attested by the finding that the use of probiotics has been associated with a reduced risk to develop illnesses with a high inflammatory background such as non-alcoholic fatty liver disease, cancer, obesity, cardiovascular diseases, or diabetes (129). Consistently, mice growing under sterile conditions had macrophages and dendritic cells unable to produce IFN-α, IFN-β, IL-6, TNF, IL-12, and IL-18 when challenged with microbial ligands, highlighting the importance of the resident microorganisms in immune system priming (130).

### 5.2. COVID-19 and microbiome dysbiosis

During SARS-CoV-2 infection, the microbiota, both at gut and lung level, faces a deep alteration in microorganism composition. In particular, the lung microbiota has been reported to undergo a diminution of the diversity and abundance of several beneficial genera which normally colonize the airways and lungs, such as *Corynebacterium, Streptococcus, Dolosigranulum, Fusobacterium periodonticum*; among them, *Dolosigranulum and Corynebacterium* were found to be significantly more abundant in COVID-19 asymptomatic subjects or those with moderate disease (131). On the other hand, several potential pathogens (such as *Pseudomonaceae, Salmonella, Serratia, Haemophilus influenzae, Moraxella catarrhalis, Prevotella, Veillonella, Staphylococcus, Peptostreptococcus, Clostridium*) appear to be enriched during COVID-19 (131); and in particular, *Prevotella salivae* was found to be a good predictor of respiratory support need in COVID-19 patients (132). Again, the amplitude of the microbial dysbiosis correlates with COVID-19 severity (133, 134).

Although SARS-CoV-2 mainly targets the respiratory system, it also affects several organs, including the digestive system. Here, the ACE2 receptor is expressed not only in the endothelial cells of the GI capillary bed, but also in the brush border of enterocytes and in gastric and colon epithelia which can be a target and replication site of the virus (135). Of note, the symptoms of COVID-19 can include nausea, diarrhea, vomiting and abdominal pain, and detection of SARS-CoV-2 in the feces occurs up to 5 weeks after the resolution of respiratory symptoms (136). Again, an association between the presence of GI symptoms, the severity of lung impairment, and the need for ventilatory support has been proposed. COVID-19 is also associated with alteration of the GI microbiota, mainly showing a diminution of the taxonomical variability of microbial species, with an increase of opportunistic or pathologic species, and a decrease of beneficial species, in particular *Ruminococcaceae and Lachnospiraceae* families (137). Some alterations, like the diminution of *Faecalibacterium prausnitzii,* inversely correlate with the severity of COVID-19 (138).

Noteworthily, a direct cause of microbiome dysbiosis could be represented by the downregulation of ACE2 operated by SARS-CoV-2 at the GI epithelial level. In fact, it has been proposed that the relative abundance of ACE2 in the GI tract is joined to ACE2 capacity to heterodimerize with amino acid transporters, warranting normal amino acid supply to enterocytes. SARS-CoV-2-dependent ACE2 deficiency could lead to an insufficient entry of tryptophan, and should in turn lead to scarce synthesis of antimicrobial peptides, affecting microbiome homeostasis and loss of epithelial integrity (135, 139). On the other hand, ACE2 downregulation can have positive consequences: Zuo et al. (138) observed that several Bacteroides species (*Bacteroides dorei, Bacteroides thetaiotaomicron, Bacteroides massiliensis,* and *Bacteroides ovatus*) are already known to be able to downregulate ACE2 expression in mice, inversely correlated with SARS-CoV-2 content in the feces of COVID-19 patients, suggesting a protective role of Bacteroides against SARS-CoV-2. An ACE2 receptor docking study suggested that ACE2 downregulation may have both positive and negative roles at the intestinal level; however, further research is needed to better characterize this dual effect.

Gut microbiota dysbiosis, typically also characterizing obesity (140), may also underpin the increased risk of COVID-19 severity observed in obese individuals (141, 142). Particularly, this increased susceptibility to develop severe symptoms in response to SARS-CoV2 infection may be dictated by the ability of gastrointestinal microbiota dysbiosis to trigger and sustain chronic inflammation (143). The latter not only typically occurs in obese individuals, but also represents one of the putative pathophysiological mechanisms linking obesity, gut microbiota dysbiosis, and COVID-19 severity (144). Indeed, obesity-related gastrointestinal microbiota dysbiosis, in concert with increased gut permeability (145), contributes to inflammation by promoting systemic endotoxemia which is the direct consequence of the leak of lipopolysaccharides (LPS) through the dysfunctional gut barrier (146). Ultimately, this inflammatory status, fueled by gut microbiota dysbiosis, amplifies the so-called “cytokine storm,” thereby predisposing subjects with obesity to more severe COVID-19 symptoms and increased risk of death (147).

Altogether, these data depict a scenario where the microbiome could act as an important environmental factor strongly contributing to the wide variability in the patient response to SARS-CoV-2. On this basis, a preventive and therapeutic approach to SARS-CoV-2 infection appears reasonable and promising, based on probiotic and prebiotic administration. The use of probiotics is aimed to positively stimulate the immunity system, reinforcing the passive barriers to prevent or reduce the possibility of viral and opportunistic pathogen entry both at lung and GI level, and to reduce the disease outcomes, preventing the cytokine storm.

### 5.3. Probiotics and prebiotics in COVID-19 treatment

The microorganism geni most frequently used as probiotics are *Lactobacillus, Bifidobacterium, Lactococcus, Streptococcus, Enterococcus, and Bacillus,* together with some strains of the genus *Saccharomycetes.* Anyway, the probiotic behavior is not linked to the whole bacterial or fungine genus. In fact, the Joint FAO/WHO Working Group Report on Drafting Guidelines for the Evaluation of Probiotics in Food in 2002 states that probiotics are “live strains of strictly selected microorganisms which, when administered in adequate amounts, confer a health benefit on the host” (148). This definition reserves the health benefit to specifically selected strains, that in addition are required to not have, nor propagate, antibiotic resistance and to be able to maintain the health benefit for the whole process of production, conservation, and distribution of the probiotic.

Probiotics improve the host’s health using the same strategies as the microbiome, but in a more effective and often targeted and detectable way. It has been clearly shown that probiotics can modulate the human microbiome and interfere with the growth of opportunistic COVID-19-related pathogens, by competing for docking sites at the epithelial surface. Furthermore, probiotics can interfere with the viral cycle, stimulating innate immunity by activating the inflammasome and the production of interferons and inflammatory cytokines which represent a first line of antiviral defense. Consistently, *Lacticaseibacillus rhamnosus GG* has been used in a neonatal mouse model of influenza as a preventive intranasal treatment. In fact, Kumova et al. showed that intranasal administration of *Lacticaseibacillus rhamnosus GG* can have immunoregulatory functions at the lung level, triggering the type-I IFN pathways via the Toll-like receptor (149).

Andrade et al. showed that, in *in vitro* systems, specific strains like *Lactobacillus plantarum* MPL16 and CRL1506, and *Dolosigranulum pigrum* 040417, increased the resistance of cultured respiratory epithelial cells to SARS-CoV-2 by inducing the production of type-I and type-III IFNs and transcription of IFN-stimulated genes, thereby potentially improving the innate antiviral response and affecting the early phases of SARS-CoV-2 infection. They noted also that the same strains could reduce cytokine production, contributing to the control of immune cell recruitment to the infection site, and subsequent of inflammatory damage. These strains appear to be promising tools to re-modulate respiratory microbiota and to counteract SARS-CoV-2 infection in the early stages (150).

Another interesting possibility is the capacity of certain probiotics to interfere with viral internalization. Silvestre Ortega-Peña et al. noted that *Staphylococcus epidermidis* is a commensal bacterium abundant in the anterior part of the nose, whose abundance is inversely correlated to serious respiratory infections. The authors not only highlighted that *Staphylococcus epidermidis* could, directly or indirectly, eliminate a wide number of pathogens, but also proposed its use as a probiotic able to prevent the development of COVID-19. Interestingly, they reported that *S. epidermidis* acts not only through the production of type-I and-III IFN pathways, but also by regulating the surface expression of the ACE2 receptor and TMPRSS2 protease (151). The importance of this finding is underlined by reports indicating that other bacteria can both release peptides able to interact with ACE2 receptors, and produce enzyme homologs to ACE2, which potentially can behave as decoy receptors for SARS-CoV-2, attenuating its entry into target cells (152).

In addition to intranasal administration, oral administration of specific probiotic strains has also been shown to be effective in the treatment of COVID-19 disease. The commercial kit Lactibiane Iki, which mixes three different strains *(Bifidobacterium lactis LA 304, Lactobacillus salivarius LA 302, and Lactobacillus acidophilus LA 201)* has been proposed to significantly reduce inflammatory markers in patients infected by COVID-19 and interstitial pneumonia (153). Another commercial product, containing three strains of *Bifidobacterium genus* and specific prebiotics, was found to reduce inflammatory markers, normalize gut microbiota composition, and increase antibody formation in 25 COVID-19 patients (154). Consistently, amelioration of antibody production is reported also by other researchers. In fact, oral administration of *Loigolactobacillus coryniformis K8 CECT 5711* to a group of healthcare workers showed a positive effect on anti-SARS-CoV-2 vaccination, leading to significantly higher antibody production after 81 days of probiotic treatment (154).

Oral administration of nisin, a food-grade peptide obtained from *Lactococcus lactis,* seems to be useful also in preventing the interaction between SARS-CoV-2 and the human ACE2 receptor. Nisin is a pentacyclic antibacterial peptide, present in several natural variants and widely used for cheese manufacturing and preservative. Several nisin variants appear to be able to efficiently interact with the ACE2 receptor and diminish SARS-CoV-2 internalization (155). As stated above, further research is needed to clarify the side effects of potential ACE2 downregulation.

**Figure 1:**
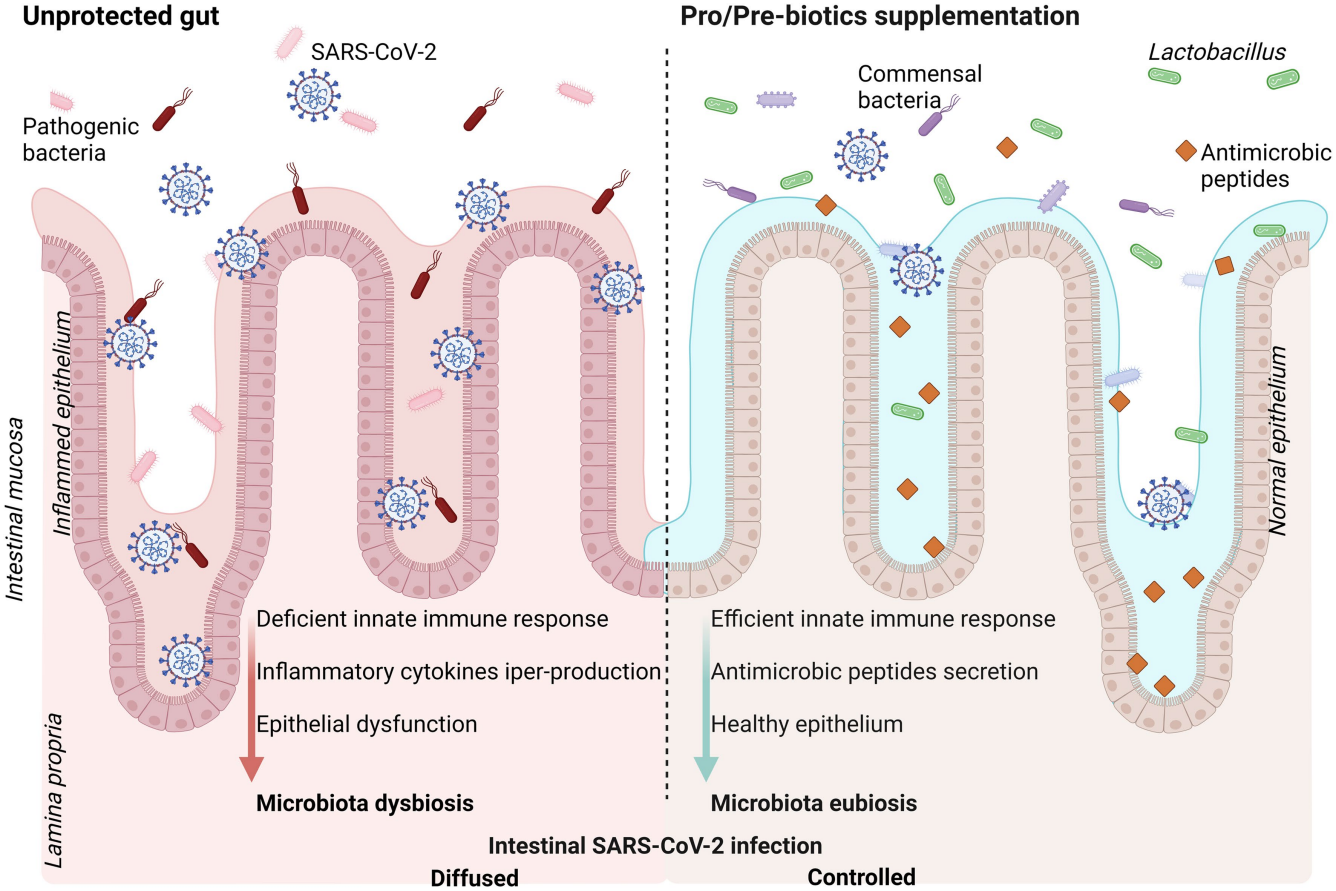
Schematic representation of intestinal mucosa exposed to SARS-CoV-2 infection (**left panel**) and positive effects of pro-and pre-biotic dietary supplementation (**right panel**). SARS-CoV-2 infection promote intestinal dysbiosis enhancing severity of the disease. Supplementation with probiotics and/or prebiotics is suggested as strategy to efficiently counteract the virus infection with better disease outcome. Created with BioRender.com.

Additionally, beneficial effects on human health can come from the administration of prebiotics (Figure 1). These are not living organisms, but represent types of not-digestible foods, which mainly include oligosaccharides, unsaturated fatty acids, dietary fibers, and polyphenols, and can be fermented by specific gut microorganisms, stimulating their growth and so reprogramming microbiome composition. Prebiotics can be beneficial when administered alone, because they can positively modify the distribution of resident microorganisms. As a note, they can also sustain the growth and survival of probiotic strains and species, with further although not easily predictable additive benefits for host health. On the other hand, large amounts of prebiotic fiber can stimulate their utilization by intestinal microorganisms, generating a large amount of gas, bloating, and discomfort.

## 6. Bioactive herbal products interfering with SARS-CoV-2 entry

Many phytochemicals with different mechanisms of action have been proposed to possess antiviral activity against SARS-CoV-2 (156). Several natural compounds, like flavonoids, steroids, coumarins, and alkaloids, were reported before the COVID-19 outbreak to possess ACE2 modulatory activity (157), encouraging this research field during the pandemic. Many studies focused on *in silico* molecular docking analysis (158–160), but relatively few molecules have been tested so far on biological systems. For this reason, in this section, we aim to resume the results obtained after assessing herbal products *in vitro* on cell line models.

Glycyrrhizic acid (GA), the main active compound of the root of *Glycyrrhiza uralensis* (licorice), was suggested as a possible candidate for COVID-19 treatment, based on its ability to reduce SARS-CoV-2 invasion by blocking the ACE2-S protein binding, as reported in human embryonic kidney 239 T cells (161). GA is also known for its anti-inflammatory properties, evidenced by the inhibition of *NF-kB* expression and cytokines secretion (162). *In vitro*, Zhao and colleagues confirmed the anti-inflammatory action of GA using an encapsulated formulation of nanoparticles to treat human monocytes (THP-1) and PBMC from healthy donors, stimulated with nucleocapsid (N) protein of SARS-CoV-2. They reported significantly reduced mRNA and protein expression of IL-1α, IL-1β and IL-6 (163).

*Stachytarpheta cayennensis,* an herbaceous plant from tropical and subtropical areas, was reported to significantly inhibit virus entry in HEK-293 T ACE2 cells (164). The characterization of the extract evidenced the presence of β caryophyllene (BCP), thymol, citral, 1,8-cineole, carvone, and limonene. BCP and limonene were previously proposed by docking studies to bind the S protein and ACE2 (160). BCP is a cannabinoid present in essential oil from common spices (e.g., cinnamon, oregano, black pepper, basil), that has been reported as one of the components with antiviral activity; however, its action tested alone was less effective compared to the total extract from *Stachytarpheta cayennensis* in countering SARS-CoV-2 S pseudovirus infection of HEK-293 T-ACE2 cells. This suggests a combined effect of different molecules in the total extract (164). BCP is also known for its anti-inflammatory proprieties, acting on different pathways including cytokine and chemokine signalling (165). Based on that, Jha and colleagues proposed BCP as a candidate for COVID-19 treatment, even if other studies are needed to verify its anti-inflammatory action (165). In agreement, limonene, the main component of essential oil from *Citrus limon,* was reported to reduce ACE2 protein levels and downregulate ACE2 and TMPRSS2 expression in colorectal adenocarcinoma cell line HT-29 (166). The same action was also shown by geranium essential oils (from *Pelargonium graveolens*), and a significant reduction of ACE2 mRNA and protein levels were also confirmed via major components: citronellol and geraniol tested as single molecules (166). Similarly, the herbal extracts of *Spatholobus suberectus dunn* (SSP) and *Polygonum cuspidatum* root and rhizome showed concentration-dependent entry inhibition in HEK293T cells (167, 168).

*Asparagus officinalis* extracts, already known for their action in counteracting breast cancer progression (169), were reported to inhibit ACE, with a positive correlation to their content in hydrophobic amino acids and gallic acid (170), suggesting a possible action also on the homolog ACE2. In addition, *Asparagus officinalis* stem extracts evidenced anti-inflammatory action through the inhibition of IL-6 and IL-1β transcription on S1-protein-stimulated macrophages (171). Inhibition of ACE2 and TMPRSS2 transcription and protein expression were also reported in 293 T cells treated for 24 h with 50 μg/mL of Theaflavin extracted from *Camellia sinensis* (172).

A molecular docking study evidenced repression of TMPRSS2 expression by withanone, a withanolide tripertenoid extracted from the root, stems, and leaves of *Withania somnifera*, a medical plant known also as Indian ginseng or winter cherry. *In vitro,* Kumar and colleagues showed that withanone causes a 40–50% reduction in TMPRSS2 expression in breast cancer cells (MCF7) (173). However, it has also been reported that this antiviral effect is associated with cytotoxicity when used at the same dose (40 μM for 48 h). In agreement, cytotoxicity was also reported in hepatocarcinoma (HepG2), breast cancer (MCF7), and normal mammary epithelium (MCF-10) cells when treated with withanone 50 μM for 72 h, while lower concentrations (20 μM) did not evidence cell viability reduction (174). For this reason, studies for possible applications for SARS-CoV-2 treatment should investigate the antiviral efficacy at a concentration of withanone that does not evidence side effects.

*Scutellaria barbata* (SB) is a widely used herb in Asia, known for its various pharmacological properties including anti-inflammatory and antiviral activities. The anti-inflammatory action of ethanol and ethyl acetate extracts of SB is sustained by phenols, flavonoids, chlorophylls, and carotenoids. These extracts significantly inhibit IL-6 and IL-1β secretion in the macrophage cell line RAW264.7 in a dose-dependent manner (175). Huang et al. reported that aqueous SB extracts, characterized by neo-clerodane diterpenoids and flavonoids followed by polysaccharides, volatile oils and steroids, inhibited the enzymatic activity of TMPRSS2 (176). In particular, 4 mg/mL of extract reduced 54.8% of TMPRSS2 protease activity. Kidney epithelial cell line Vero E6, which express high ACE2 and low TMPRSS2 levels, and human lung carcinoma cells Calu-3, which express high TMPRSS2 levels, were used as cell models to test SB activity. Pre-treatment with SB extract and then infection with SARS-CoV-2 pseudovirus reduced infection in Calu-3 cells but not in VeroE6, suggesting that SB affects TMPRSS2, ultimately reducing virus entry (176).

The same cellular models were used also by Kim et al. (177) to demonstrate that platycodin D (PD), a glycosylated triterpenoid saponin extract from the root of *Platycodon grandiflorum,* inhibited virus entry both in Calu-3 (TMPRSS2-high/positive) and Vero E6 cells (TMPRSS2-low/negative). In this case, the mechanism is still unknown; however, the authors suggested that PD interferes with virus entry by interacting with cholesterol and preventing virus fusion to the host cells. They demonstrated that cholesterol depletion in host cells decreases 2.5 times the PD effects, supporting the hypothesis that cholesterol facilitates PD action in host cells (177). The same research group, taking advantage of these results, identified the three chemical groups presented on the PD structure that were essential for inhibition of virus entry into the cells, to develop new synthetic saponins. The new molecules efficiently inhibit the fusion to the ACE/TMPRSS2-positive cells (H1299, lung carcinoma cells) with a 2-fold increase in potency compared to the initial natural compound (178). Additionally, phenolic components from *Platycodon grandiflorum* extracts were reported to possess anti-inflammatory activity by reducing IL-6 and TNF-α production in LPS-stimulated macrophage cell lines (RAW 264.7) (179), suggesting multiple bioactive components in the total extract could be useful for COVID-19 treatment.

Similarly to saponin PD, even for astersaponin I (AI), a triterpenoid saponin in *Aster koraiensis*, the observed inhibition of SARS-CoV-2 infection was dependent on effects on cholesterol (180). In this work, the authors demonstrated that treatment with AI induces increasing cholesterol content in the cell and in the endosomal membranes, and that this interferes both with the entry of the virion as well as with syncytium formation. Results of fusion experiments performed in H1299 cells demonstrated that 5 μM of AI inhibited entry and prevented syncytia of more than 90%. The effects were shown for wild-type and D614G variant SARS-CoV-2 and led authors to propose AI as a broad-spectrum agent also against other enveloped viruses.

Following a similar reasoning, epigallocatechin gallate (EGCG), the green tea catechin, has been proposed as a future pan-coronavirus attachment inhibitor due to its effects on cell-surface glycans, as demonstrated by LeBlanc and Colpitts (181). In their work, EGCG treatment of Huh7 and A459 inoculated with seasonal human CoVs, HCoV-229E and HcoV-OC43, inhibited infectivity at low micromolar concentrations (IC_50_ < 1 μM), with minimal effects on cell viability. Furthermore, they showed that EGCG was able to inhibit entry of SARS-CoV-1, SARS-CoV-2 and its delta and omicron variants, and WIV1-CoV (a bat coronavirus able to bind human ACE-2) with 15 μM < IC_50_ < 25 μM. Finally, they demonstrated that the antiviral effect of EGCG was caused by the heparan sulfate blocking of virions binding to the cell membrane, with the same mechanism as heparin. Considering that interactions with membrane glycans are shared by many viruses to initiate the infection, and that heparan sulfate proteoglycans are necessary for SARS-CoV-2, the authors conclude that EGCG can be an efficient antiviral, but its low stability and rapid metabolism are important limitations. Nonetheless, other authors have observed a partial reduction of SARS-CoV-2 replication *in vivo* in C57BL/6 mice infected intranasally and treated orally with 10 mg/kg daily of EGCG for 2 weeks (182), indicating a possible future application. Other studies have focused on EGCG and ACE2, observing inhibition of S protein binding to ACE2 receptor by neutralization (183), ELISA (184), and entry or infectivity (184, 185) assays, testing EGCG in the range 0–100 μM on several cellular models. Interestingly, the work of Liu et colleagues reported an efficient reduction of infection when using live SARS-CoV-2 and HcoV-OC43 viruses on Calu-3, HEK-293 T-ACE, and HCT-8 cells, indicating that pre-treatment is fundamental for a significant effect (185). Collectively, these results indicate that EGCG can work both specifically on ACE2, as well as via unspecific interference with heparan sulfates.

Another phenolic compound that was reported to reduce SARS-CoV-2 infection in TMPRSS2-negative Vero E6 cells was the resveratrol tetramer hopeaphenol, a compound extracted from various plants including *Hopea, Vitis,* and *Shorea,* that was suggested to be acting without affecting TMPRSS2. Further studies are required to elucidate its mechanism of action (186).

An herbal mixture called virofree, containing active compounds including quercitin, hesperidin, genistein, daidzein, and resveratrol, was reported to repress protein S binding to ACE2 by *in vitro* biochemical-binding ELISA assay. Tested on Calu-3 cells, virofree dose-dependently decreases the protein expression of ACE2 and TMPRSS2, suggesting an antiviral activity through inhibition of virus entry into the cells (187).

**Figure 2:**
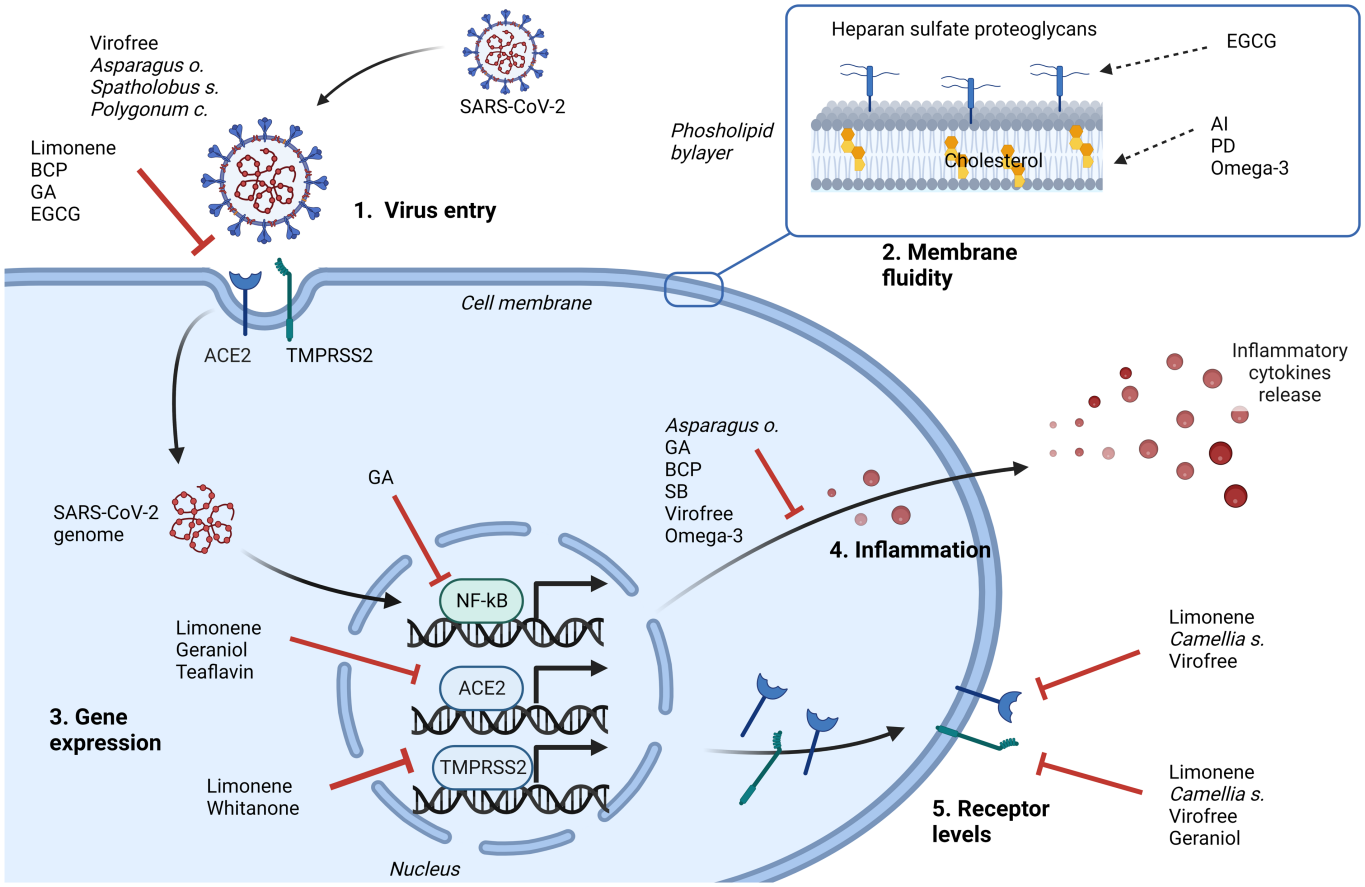
Representation of the major cellular events that can be inhibited by natural products in contrasting SARS-CoV-2 infection. Their effects can limit the virus entry, directly interfering with cellular receptor binding (1), or indirectly changing the membrane fluidity (2), and/or can affect some pathways driven by virus. Intracellularly, involved mechanisms can be the downregulation of *NF-kB*, *ACE2* or *TMPRSS2* gene expression (3), the inhibition of cytokines release (4), to control the inflammatory process, and the downregulation of ACE2 and/or TMPRSS2 protein levels (5). AI, astersaponin I; BCP, β caryophyllene; EGCG, epigallocatechin gallate; GA, glycyrrhizic acid; PD, platycodin D; SB, *Scutellaria barbata*. Created with BioRender.com.

Overall, many phytochemical compounds present in herbs or herbal extracts might be potentially used for relieving SARS-CoV-2 infection, limiting virus entry, or reducing inflammation (Figure 2). However, the identification of bioactive compounds in extract mixtures, the efficient non-toxic antiviral range of concentrations, and the specific mechanisms of action remain as open questions in many cases; thus, further *in vitro* and *in vivo* deepening are needed before moving to clinical trials investigations.

Furthermore, innovative approaches considering the use of nanoparticles to ameliorate the bioavailability of the investigated compound could reconsider some molecules and improve *in vivo* results.

## 7. Conclusion

The COVID-19 pandemic appears to be a global threat unfortunately lacking robust medical treatments. Several tools have been used to manage COVID-19 symptoms and the clinical aftermath of this illness, including old and new antiviral drugs, and plasma from convalescent patients or purified antibodies. But the most promising medical approach relies upon massive vaccination. Anyway, despite the success of vaccine trials and the presence of different vaccine platforms, several concerns exist, reducing vaccine massive utilization and limiting their efficacy. Social concerns, like logistic and economic issues, together with vaccine hesitancy, can severely limit the dissemination of vaccination. In addition, the quite-fast appearance of new virus variants, together with the wide variability of genetic, health and nutritional status of human beings, can influence the severity of viral illness and consequently interfere with the effectiveness of vaccination in protecting people from severe aftermath of viral infection. In a similar scenario, it seems reasonable that COVID-19 therapeutics can include some supplementary nutritional approaches. In particular, vitamins and nutrients, like vitamins A, E and D, other polyunsaturated lipids and minerals like zinc, can be lowered in at least some part of the population, such as the elderly, or patients suffering from long-lasting subclinical or full-blown inflammatory and oxidative conditions, e.g., arthritis, obesity, diabetes, hypertension, cardiopathies, and cancer. Adequate nutrient supplementation could not only boost the immune system, but also has been shown to prevent viral entry, as reported above, interfering with the process of membrane fusion subsequent to ACE2 docking and TMPRSS2 action.

Supplementation of prebiotics and probiotics has been largely shown to strongly prompt and reshape the immune system, ultimately also ameliorating antibody production and vaccine effectiveness. Similarly, herbal-derived compounds or extracts from herbal mixtures offer a wide panel of immunoactive substances able to sustain the anti-viral response. An interesting feature, shared by probiotics and herbals, is represented by the ability to reduce viral entry, preventing SARS-CoV-2 infection. The entry strategy implicates several interactions with the cell surface, including S protein priming, ACE2 binding and membrane fusion, or, in absence of TMPRSS2 activity, vesicle-mediated endocytosis. Independent of the exact mechanism for viral RNA liberation into the cytoplasm, interaction with the ACE2 protein appears to be the crucial event for viral infection. Of note, extracts from licorice, *Stachytarpheta cayennensis, Spatholobus suberectus dunn* (SSP) and *Polygonum cuspidatum* have been shown *in vitro* to inhibit virus entry, in particular blocking receptor docking or downregulating ACE2 expression. On the other hand, Theaflavin extracted from *Camellia sinensis* and withanone from *Withania somnifera* have been shown to reduce the level of TMPRSS2, while other plants like as *Scutellaria barbata* are effective in reducing TMPRSS2 activity, and consequentially, S protein priming. This latter finding is of particular interest, because TMPRSS2 activity appears to be higher in the respiratory airways and lungs where SARS-CoV-2 exerts its main infectious effects. Also, several strains of probiotics have been shown to reduce viral infection, through both nasal and oral administration. They can act through different but often complementary mechanisms. Probiotics can specifically interfere with viral access to the cell surface, and/or produce peptides able to reduce the interaction between virus and ACE2, like nisin and its derivatives. Decrease of the surface expression of ACE2 receptor and TMPRSS2 protease appears to be an important tool in probiotic antiviral activity. Concurrent triggering of immune reactions represents an important tool in antiviral defense, although IFN activation, which represents a branch of the antiviral action, can paradoxically increase ACE2 expression. This occurrence can in principle increase virus replication, but at the time, evidence for an IFN-mediated increase in COVID-19 severity is lacking; in addition, it has been reported that interferons induce a truncated isoform of ACE2 not supporting virus replication (188). On the other hand, with ACE2 having a role in amino acid transport, at least in the intestine and kidney, its downregulation can potentially imply undesired side effects. This urges further research to clarify all the positive and negative implications of potential ACE2 downregulation.

Lastly, polyunsaturated lipids can alter membrane structure at lipid rafts where ACE2 is localized, thereby influencing viral entry. Of note, several lipids, including polyunsaturated omega-3 fatty acids, linolenic acid, and eicosapentaenoic acid, can directly interfere with virus-binding to ACE2, thereby significantly reducing viral entry. In addition, several bioactive herbal products, including saponins, such as triterpenoid platycodin D and astersaponin I, act on the cholesterol content of lipid rafts, interfering with viral internalization routes. In a similar way, the green tea catechin epigallocatechin gallate can inhibit viral binding to cell surface glycan and ACE2.

New Omicron variants seem to prefer the endocytosis pathway to TMPRSS2 /ACE2 and membrane fusion (189). Again, further research is needed to explore the contribution of different entry routes for each viral variant. These caveats notwithstanding, the world of natural products represents a huge *reservoir* of biochemical and biological variability, that appears wide enough to offer efficient and hopefully decisive tools to cover, in the general population, the need to counteract viral entry in all its different forms.

## Author contributions

GZ, DS, MV, AR, RV, and MP: conceptualization, revision process, and final editing. GZ, DS, MV, AR, RV, MP, EZ, GL, and LC: writing – original draft preparation and writing – review and editing. All authors contributed to the article and approved the submitted version.

## Funding

This work was funded by funding from the FIR 2021 to RV.

## Conflict of interest

The authors declare that the research was conducted in the absence of any commercial or financial relationships that could be construed as a potential conflict of interest.

## Publisher’s note

All claims expressed in this article are solely those of the authors and do not necessarily represent those of their affiliated organizations, or those of the publisher, the editors and the reviewers. Any product that may be evaluated in this article, or claim that may be made by its manufacturer, is not guaranteed or endorsed by the publisher.
